# Optimized pipeline and designer cells for synthetic-biology-based high-throughput screening of viral protease inhibitors

**DOI:** 10.1016/j.crmeth.2025.101139

**Published:** 2025-08-07

**Authors:** Shlomi Edri, Shayma El-Atawneh, Tehila Ernst, Maayan Elnekave, Chaja Katzman, Tali Lanton, Ido Aldar, Omri Wolk, Noa Stern, Amiram Goldblum, Lior Nissim

**Affiliations:** 1 Department of Biochemistry and Molecular Biology, Institute for Medical Research Israel-Canada, Faculty of Medicine, The Hebrew University of Jerusalem, Jerusalem 91120, Israel; 2 Laboratory of Molecular Modeling, Institute for Drug Research, Faculty of Medicine, The Hebrew University of Jerusalem, Jerusalem 91120, Israel

**Keywords:** synthetic biology, synthetic gene circuits, biotechnology, biomedicine, antiviral drugs, functional drug screen, high-throughput drug screen, COVID-19, SARS-CoV-2, 3CLpro

## Abstract

A reliable, efficient, high-throughput pipeline to evaluate viral protease inhibitors would enhance antiviral drug discovery. Methods such as crystallography and phenotypic screening are often constrained by complex assay conditions, limited physiological relevance, or live virus handling safety concerns. Proof-of-concept studies previously demonstrated synthetic gene circuits that produce a quantitative reporter upon protease inhibition, enabling functional virus-independent evaluation of viral protease inhibitors in live cells. Using the SARS-CoV-2 3-chymotrypsin-like protease (3CLpro) as a model, we advanced this approach into a high-throughput first-pass qualitative assay (“hit/no-hit”) to rapidly identify promising drug candidates. Our optimized circuit design was used to produce stable HEK293T and HeLa designer cells that generate two distinct fluorescence outputs, simultaneously reporting protease inhibition and cytotoxicity. The screening pipeline is designed to minimize labor, costs, and false-positive observations, thus enabling versatile, safe, and efficient functional drug screening suitable for any conventional biological laboratory.

## Highlights

• Designer cells enable safe, scalable, and high-throughput antiviral drug screening
• Synthetic circuits quantify protease inhibition and cytotoxicity in live cells
• Dual-fluorescence outputs simplify screening and minimize false positives
• Modular platform adaptable for various protease targets beyond SARS-CoV-2

## Motivation

Current methods to evaluate viral protease inhibitors, such as crystallography and phenotypic screening, are often constrained by complex assay conditions, limited physiological relevance, or live virus handling safety concerns. Synthetic gene circuits that evaluate these have been previously demonstrated both *in vitro* and *in vivo*. A reliable, efficient, high-throughput pipeline using enhanced synthetic gene circuits and stable designer cells for fluorescence-based quantitative measurement to evaluate viral protease inhibitors would enhance antiviral drug discovery.

## Graphical abstract

**Figure:**
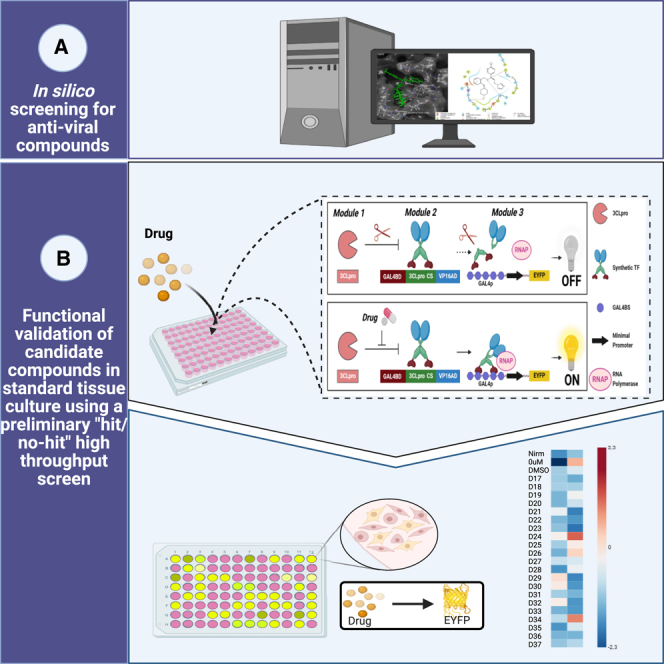

## Teaser

Edri et al. present a modular synthetic biology platform for high-throughput screening of viral protease inhibitors in live cells. Using designer HEK293T and HeLa cells engineered with optimized gene circuits, their dual-fluorescence assay enables the safe, cost-effective, and scalable detection of protease inhibition and cytotoxicity, thereby minimizing false positives and facilitating the discovery of antiviral drugs.

## Introduction

Despite global efforts, viral-borne diseases remain a significant risk to public health.^1^ Vaccines provide preventive care against viral threats but must be administered before infection, could lose efficacy against emerging variants, frequently require booster shots, and are ineffective in immunosuppressed individuals. Antiviral drugs could provide effective post-infection treatment even for immunosuppressed patients, but their discovery remains challenging. Viral proteases, such as the main SARS-CoV-2 3-chymotrypsin-like protease (3CLpro), are attractive drug targets since they are essential for the viral life cycle and typically lack homologous counterparts in human cells.^2^^,^^3^^,^^4^^,^^5^ Therefore, a straightforward, reliable, high-throughput pipeline to identify viral protease inhibitors could impact public health.

Computational technologies enable the virtual identification of candidate antiviral compounds by methods such as drug repurposing, virtual screening of molecule libraries, *de novo* design of small molecules, and optimization of known viral inhibitors.^6^^,^^7^^,^^8^ These predictions are experimentally validated by methods including crystallography, fluorescence resonance energy transfer (FRET), mass spectrometry, and phenotypic screening.^9^^,^^10^^,^^11^^,^^12^ However, these approaches are often limited by the lack of easily measurable markers for inhibition efficiency, non-physiological assay conditions, or the necessity for high-containment laboratories for assays involving live viruses. Therefore, high-throughput, functional, safe, straightforward, and cost-effective cell-based methodologies to evaluate candidate compounds would enhance drug discovery. Cell-based reporter assays were previously developed to determine protease inhibitor efficiency in live cells, including FlipGFP,^13^^,^^14^^,^^15^ luciferase complementation assay,^16^ Protease-Glo luciferase,^15^^,^^17^ HIF1α oxygen-dependent degradation domain (ODD)-luciferase,^18^ and replicon systems.^19^^,^^20^ However, optimizing engineered proteins is complicated, and these assays often do not directly measure compound-mediated cytotoxicity, which can skew the screening results. Therefore, flexibility in tuning assay sensitivity and replacing output genes while accounting for cytotoxicity is essential.

Synthetic biology focuses on engineering artificial biological parts, devices, and systems with novel functions as powerful tools for fundamental research, medicine, and biotechnology.^21^^,^^22^^,^^23^^,^^24^^,^^25^ Synthetic gene circuits have been designed for diverse applications, including the precise targeting of cancer cells, enhancement of immunotherapies, treatment of various diseases, programming of cellular behavior, and studying of biological systems.^24^^,^^25^^,^^26^^,^^27^ Tunable autoproteolytic gene switches (TAGS), designed to estimate the inhibition of various viral proteases *in vitro* and *in vivo,* provide superior performance and easy tunability.^23^ TAGS tailored to evaluate inhibition of 3CLpro include a 3CLpro fused to a synthetic transcription factor (synTF) that contains a 3CLpro cleavage site (CS). The protease cleaves the CS and thereby deactivates the synTF.^23^ Therefore, upon effective 3CLpro inhibition, the synTF is stabilized and activates a synthetic promoter comprised of tandem GAL4 binding sites upstream of a minimal adenoviral promoter (GAL4p), which regulates luciferase expression. Consequently, following TAGS transfection into model cells, luciferase levels provide a direct quantitative readout of 3CLpro inhibition. This versatile approach accounts for cellular drug uptake and protease function in relevant physiological conditions. However, transfecting cells before each experiment is labor intensive and may lead to measurement fluctuations due to variability in transfection efficiency. Additionally, incorporating an easily measurable cytotoxicity indicator would improve the screening process.^13^^,^^14^^,^^15^ Finally, luciferase measurements require the addition of a suitable substrate, which complicates sample preparation and increases screening costs.

Replacing luciferase with a fluorescent gene would overcome these limitations but at the cost of reduced sensitivity, which could potentially overlook lower inhibition efficiencies. Fluorescence measurements provide closer linear correlations between output levels and protease inhibition, enabling more direct quantification of inhibitor efficiency.^28^ In comparison, luciferase-based assays are typically 10- to 100-fold more sensitive due to signal amplification by enzymatic luciferase activity.^28^ Nevertheless, enhanced sensitivity may become a disadvantage in drug discovery, as it can lead to false-positive identification of compounds that exhibit measurable protease inhibition but lack sufficient potency for therapeutic efficacy. Given the simplicity-sensitivity trade-off, fluorescent protein outputs are more suitable for first-pass screening pipelines.

To this end, we further enhanced the screening process by systematically optimizing the TAGS design, employing fluorescent protein output, integrating a cytotoxicity indicator, and generating stably transduced designer cells to improve inter-experimental consistency. Our experimental pipeline evaluates protease inhibition, provides a measurable estimation of cytotoxicity, and efficiently excludes false-positive observations. Specifically, we generated designer HEK293T and HeLa cells stably transduced with an optimized design that generates an enhanced yellow fluorescent protein (EYFP) output. These cells also express enhanced cyan fluorescent protein (ECFP) to account for drug-mediated cytotoxicity.^29^^,^^30^ The resulting screening protocol, suitable for both flow cytometry and plate reader measurements, was implemented to evaluate 97 candidate compounds predicted by molecular docking to inhibit 3CLpro.^31^ The designer cells and screening pipeline are readily available for use in standard laboratories. Finally, our modular design could be adapted to screen for drugs targeting other viral proteases, as previously described.^23^

## Results

### Circuit design and optimization

**Figure 1:**
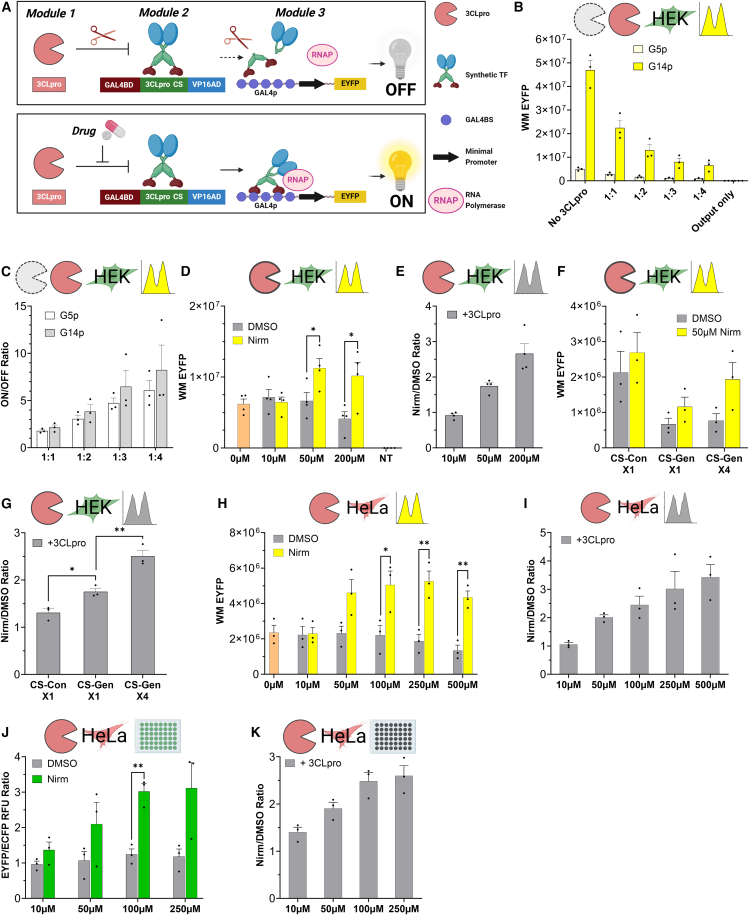
Design and optimization of synthetic gene circuits for 3CLpro inhibitor screening (A) The platform consists of three genetic modules encoded on lentiviral vectors. Module 1: a constitutively active promoter drives the expression of the viral protease 3CLpro. Module 2: a constitutively active promoter drives the expression of a chimeric transcription factor comprising the yeast GAL4 DNA-binding domain (GAL4BD) fused to a 3CLpro cleavage site (CS) substrate and a subsequent VP16 transcription activation domain (VP16AD). Module 3: the synthetic GAL4p, comprising GAL4 binding site repeats (GAL4BSs) encoded upstream to a minimal promoter, is activated by the synthetic transcription factor and regulates the expression of an enhanced yellow fluorescent protein (EYFP). Upper panel: 3CLpro cleaves the chimeric transcription factor at its cognate CS, thereby downregulating EYFP expression. Lower panel: in the presence of a 3CLpro inhibitor, the synthetic transcription factor remains intact, resulting in increased EYFP expression. (B and C) Impact of the number of GAL4BS repeats and 3CLpro concentration on the circuit output. Cells were transduced with module 2 and different module 3 versions in which the GAL4p comprises 5 or 14 GAL4BSs. Subsequently, the cells were transduced with four different dilutions of lentivirus encoding module 1. The *x* axis represents the ratio of lentivirus concentrations encoding module 2 (synthetic transcription factor) to those encoding module 1 (3CLpro) used for transduction. Thus, lower ratios (e.g., 1:1) correspond to lower 3CLpro concentrations in the transduced cells, and higher ratios (e.g., 1:4) correspond to higher 3CLpro concentrations. Results for each sample are presented as (B) the weighted median (WM) of EYFP fluorescence and (C) the ratio of output generated in cells lacking 3CLpro (ON) to the output in cells expressing 3CLpro (OFF). (D and E) EYFP output levels generated by the circuit following 3CLpro inhibition by nirmatrelvir (Nirm) in HEK293T cells engineered with all three circuit modules. (D) The WM of EYFP fluorescence was measured by flow cytometry, and unpaired t tests were performed to compare EYFP expression levels between Nirm-treated samples and their corresponding DMSO controls for each drug concentration. (E) Circuit sensitivity, defined as the ratio of fluorescence generated by a Nirm-treated sample to the fluorescence generated by the corresponding DMSO-treated control. (F and G) Optimization of 3CLpro CSs in module 2. Cells were transduced with module 2 variants in which the synthetic transcription factor contains either a single repeat of the consensus CS sequence AVLQSGFR (CS-Con x1), a single repeat of the synthetic general CS sequence VARLQSGF (CS-Gen x1), or four tandem repeats of the synthetic general CS sequence (CS-Gen x4). (F) The WM of EYFP fluorescence was measured by flow cytometry. (G) Circuit sensitivity for all module 2 variants. Unpaired t tests were performed to compare the Nirm/DMSO ratio among the different CS groups. (H and I) EYFP output levels generated by the circuit following 3CLpro inhibition by Nirm in HeLa cells engineered with all three circuit modules. Results are presented as (H) The WM of EYFP fluorescence, measured by flow cytometry, and unpaired t tests performed to compare EYFP expression levels between Nirm-treated samples and their corresponding DMSO controls for each drug concentration. (I) Circuit sensitivity in HeLa cells. (J and K) Plate reader measurements of 3CLpro inhibition by Nirm in HeLa cells engineered with all three circuit modules. Results are presented as (J) The ratio of EYFP relative fluorescence units (RFU) to ECFP RFU. Unpaired t tests were performed to compare EYFP/ECFP ratios between Nirm-treated samples and their corresponding DMSO controls for each drug concentration. (K) Circuit sensitivity in plate reader measurements. Data are presented as the mean of biological replicates, with individual dots on each bar representing replicate values. Error bars indicate the standard error of the mean (SEM); *n* = 3 or 4 biological replicates as indicated by the number of dots (∗*p* < 0.05, ∗∗*p* < 0.01).

The TAGS design was enhanced by several modifications. First, the 3CLpro-synTF fusion protein was separated into two independent proteins. This enabled adjustments of 3CLpro:synTF ratios to tune circuit sensitivity. Subsequently, the luciferase output was replaced with EYFP. The resulting circuit consists of three genetic modules, encoded on separate lentiviral vectors (Figure 1A). Module 1 comprises the constitutively active human ubiquitin C promoter (hUbCp), regulating 3CLpro expression. Module 2 includes the constitutively active human SSX1 promoter (SSX1p), regulating a synTF comprising the yeast GAL4 DNA-binding domain (GAL4BD) fused to the viral VP16 transcription activation domain (VP16AD) via a 3CLpro CS.^14^^,^^23^^,^^32^^,^^33^ Module 3 consists of a synthetic promoter (GAL4p) that is activated by the synTF,^32^ regulating the expression of EYFP (GAL4p-EYFP). Consequently, 3CLpro constantly deactivates the synTF by cleaving the CS domain, thereby inhibiting EYFP production. Conversely, in the presence of a 3CLpro inhibitor, the synTF remains intact and induces EYFP expression.

We initially systematically optimized the circuit design to enhance the screening sensitivity, defined as the ratio of EYFP levels in untreated control designer cells compared with EYFP levels in designer cells treated with an efficient 3CLpro inhibitor. First, we examined how modifications to the GAL4p tune the circuit response. GAL4p comprises tandem repeats of GAL4 binding sites (GAL4BS) encoded upstream of a minimal promoter.^32^ Since the number of GAL4BS repeats determines the GAL4p dynamic range and activation threshold,^26^ we compared outputs from GAL4p variants containing 5 or 14 GAL4BSs (G5p and G14p, respectively). To this end, HEK293T cells were sequentially transduced with lentiviruses encoding module 2, followed by module 3 containing either G5p-EYFP or G14p-EYFP. To evaluate the circuit response across a range of 3CLpro:synTF ratios, these cells were transduced with varying concentrations of lentivirus encoding module 1. In both designs, EYFP levels measured by flow cytometry consistently decreased as 3CLpro levels increased. However, G14p generated approximately 10-fold higher outputs than G5p at equivalent 3CLpro:synTF ratios (Figure 1B). Both designs exhibited comparable ON/OFF fold changes, defined as the output levels with 3CLpro (ON) versus without 3CLpro (OFF; Figure 1C). We subsequently selected G5p at high 3CLpro:synTF ratios to minimize background EYFP expression and prevent signal saturation. Nevertheless, G14p could be used for experimental configurations with higher output detection thresholds, providing flexibility in selecting the output type and measurement device.

Next, we evaluated the circuit performance with varying concentrations of nirmatrelvir, a 3CLpro inhibitor that is part of the FDA-approved drug Paxlovid.^34^ A concentration of 50 μM nirmatrelvir dissolved in DMSO yielded optimal circuit sensitivity with minimal cytotoxicity (Figures 1D and 1E), without increasing output levels in control cells lacking 3CLpro (Figures S1A and S1B). Additionally, EYFP output plateaued at 100 μM and decreased at 500 μM, consistent with expected drug saturation kinetics and normalization artifacts at cytotoxic concentrations. Using these settings, we refined additional parameters of the experimental protocol, including incubation durations and drug/media refreshment (Figures S1C–S1F). The 5-day setup provided optimal sensitivity and a sufficient time frame to observe cytotoxicity, both of which are critical for compound evaluation. This setup was therefore employed in subsequent experiments.

Finally, circuit sensitivity was tuned by replacing the previously described CS domains in module 2,^23^ optimized for cleavage by SARS-CoV,^35^ with a synthetic general CS optimized for cleavage by 3CLpro from all coronavirus groups (CS-Gen).^33^ This modification may also broaden the utility of the circuit for additional virus types. For reference, CS-Gen was also compared to a native consensus CS (CS-Con).^16^ A single CS-Gen provided better sensitivity than a single CS-Con (∼1.3 vs. 1.75-fold; Figures 1F, 1G, S1G, and S1H). Multiple tandem CS repeats can enhance cleavage probability, thereby improving the circuit dynamic range and sensitivity. Accordingly, a design including four CS-Gen repeats provided the highest sensitivity and was therefore selected for subsequent application (∼2.5-fold; Figures 1F, 1G, S1G, and S1H). Thus, circuit sensitivity could be optimized by modifying the composition and number of CS repeats, thereby providing additional flexibility for future applications.

### Optimization of the screening pipeline

Since sample preparation for flow cytometry is time consuming and labor intensive, the pipeline was adapted for plate reader measurements, suitable for automated screening systems. This required several modifications to the experimental setup. HEK293T cells adhere poorly to culture plates, causing cell loss during media refreshment and sample wash. HeLa cells, which adhere significantly better, were therefore used instead. HeLa designer cells engineered with the circuit were further transduced to stably express ECFP. Since ECFP levels are proportional to the number of live cells per well, this signal provides a direct quantitative estimate of compound-mediated cytotoxicity, enabling normalization of the EYFP output to cell count.

Circuit sensitivity for HeLa and HEK293T designer cells was comparable, yielding a ∼2- to 3.5-fold change in both flow cytometry (Figures 1H, 1I, S2A, and S2B) and plate reader measurements (Figures 1J, 1K, S2C, and S2D). These results demonstrate the reliability of this approach across multiple cell lines and robust qualitative circuit performance over a broad range of drug concentrations, including cytotoxic levels up to 500 μM. To further facilitate plate reader-based ECFP measurements as an estimation of compound-mediated cytotoxicity, we characterized the number of live cells as a function of ECFP levels per well (Figure S2E). For this purpose, HeLa designer cells were plated with increasing DMSO concentrations, including cytotoxic levels. ECFP levels per well were then measured using a plate reader, after which cells were collected, and live cell counts were performed using an automated cell counter. These data demonstrate a tight correlation between ECFP reads and live cell count, further confirming ECFP measurements as a marker of cytotoxicity. However, certain compounds may alter ECFP expression independently of their cytotoxic activity. Therefore, cell viability and cytotoxicity should be further evaluated using additional assays in downstream experiments.

Once established, the plate reader setup was used to examine 97 candidate inhibitors identified by virtual docking of approximately two million compounds to the crystal structure of 3CLpro.^36^ Selection was restricted to candidates forming a salt bridge with Cys145, rather than the covalent bond formed by the known 3CLpro inhibitors N3 and nirmatrelvir.^36^^,^^37^ We initially filtered the Enamine HTS dataset^38^ based on interaction filtration,^39^ yielding 328 candidates (table in the Data S1 file, which includes raw data and cell line composition). From these, we selected the top candidates with a total score (TS) ≥ 4, along with additional molecules that met at least one criterion regarding the docking score, buried surface area (BSA), number of contacts, or rotatable bonds, resulting in 145 compounds (table included in the data file Data S1). Of these, 97 compounds available in stock were purchased. Ideally, each compound would have been screened at IC_50_ concentrations, but to minimize labor for this first-pass qualitative screen, individual IC_50_ values were not determined. Instead, the screen was conducted at 50 μM and 100 μM, the highest DMSO concentrations causing minimal cytotoxicity (Figures 1D and 1H), knowing that these concentrations may not reflect the IC_50_. This compromise is acceptable only for preliminary first-pass qualitative assays that provide hit/no-hit predictions. However, quantitative IC_50_ values should be determined in follow-up assays designed for dose-response assessment, and subsequent evaluation of candidate compounds should be performed at IC_50_ concentrations.

**Figure 2:**
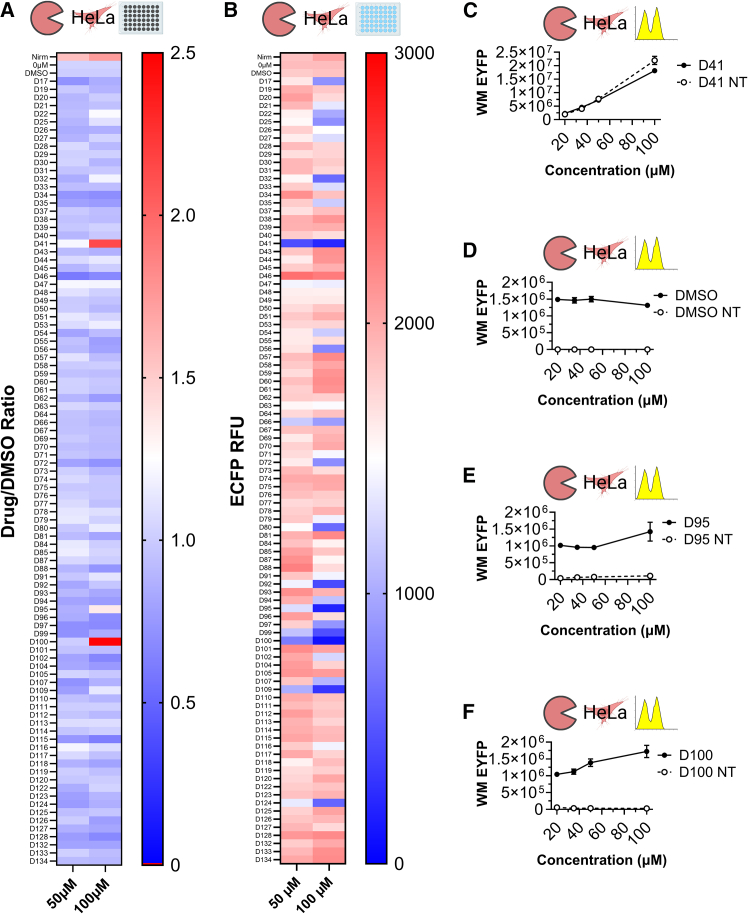
Pipeline optimization of the high-throughput drug screen (A and B) Screening of 97 candidate 3CLpro inhibitory compounds in HeLa cells using plate reader measurements. Drug concentrations are indicated at the bottom of each heatmap. Heatmaps represent (A) Potential 3CLpro inhibition by each compound. EYFP/ECFP ratios were determined for each sample. Potential 3CLpro inhibition was estimated by calculating the ratio of EYFP/ECFP values of each compound-treated sample, normalized to the EYFP/ECFP ratio of the corresponding DMSO control. (B) Compound-mediated cytotoxicity estimated by ECFP relative fluorescence units (RFUs). (C–F) The EYFP signal generated by HeLa cells, either naive (NT) or engineered with all three circuit modules, following treatment with candidate compounds. Results presented as the weighted median (WM) of EYFP fluorescence measured by flow cytometry. Data are presented as the mean of biological replicates. Error bars indicate the standard error of the mean (SEM); *n* = 3 biological replicates.

Compounds 41, 95, and 100 demonstrated potential 3CLpro inhibition (Figures 2A and S3A). However, overall ECFP levels in these samples were low and decreased with increasing compound concentrations (Figure 2B), suggesting that cytotoxicity may have skewed the measurement results, determined by EYFP:ECFP ratios. Additionally, autofluorescent compounds that penetrate cells may not be effectively removed by washing, causing false-positive inhibition results. To examine this hypothesis, these candidate compounds were further evaluated using flow cytometry, which does not require the ECFP signal to account for cell number. To rule out compound-mediated autofluorescence as the source of the EYFP signal, the EYFP output was also measured in compound-treated naive cells that were not transduced with the circuit (Figures 2C–2F, S3B, and S3C). For compound 41, EYFP fluorescence was similar in both designer and naive cells, indicating that the signal originated from the compound rather than from 3CLpro inhibition (Figures 2C and S3B). For all other compounds, EYFP levels were significantly higher in designer cells than in naive cells (Figures 2D–2F). However, no significant differences were observed between the DMSO controls and the treated groups, indicating that these compounds did not effectively inhibit 3CLpro (Figure S3C).

These data emphasize the importance of validating plate reader results with follow-up flow cytometry measurements in both naive and designer cells to avoid false-positive inhibition observations. An alternative approach to preclude false-positive results caused by compound-mediated autofluorescence is to include naive cells in the initial plate reader screen, which otherwise involves only designer cells. This strategy would reduce the overall experimental turnaround time but would also double the number of samples in the initial screening phase, significantly increasing the workload. Accordingly, the screen should be configured to prioritize either labor minimization or shorter screening duration, depending on resource constraints. Finally, fluorescence from tested compounds could leak into the ECFP channel, leading to false-negative results due to artificially reduced EYFP:ECFP ratios. In all plate reader samples (Figure 2), ECFP levels in compound-treated samples remained within ∼1.2-fold of ECFP measured in the corresponding DMSO controls, precluding significant compound-mediated autofluorescence leakage into the ECFP channel. Nevertheless, ECFP levels in all samples must be compared to the corresponding control to avoid false-negative results.

## Discussion

Viral proteases are favorable targets for antiviral drugs. Computational prediction platforms can identify candidate protease inhibitors^6^^,^^7^^,^^8^ but require experimental validation. Methods such as crystallography,^9^ mass spectrometry,^10^ and FRET^11^^,^^12^ can determine inhibitor-protease binding. However, these methods involve complex procedures performed under non-physiological conditions and do not provide functional inhibition data in a physiological context. Phenotypic screening addresses this limitation but often requires handling live viruses.^10^ Synthetic gene circuits are superior to protein engineering-based virus-free approaches in terms of performance, flexibility in output selection, sensitivity, dynamic range, and adaptability to additional viral targets.^13^^,^^14^^,^^16^^,^^23^^,^^40^

To this end, we developed an experimental pipeline for efficient high-throughput functional screening of candidate 3CLpro inhibitors, based on designer cells engineered with synthetic gene circuits. Upon 3CLpro inhibition, the circuits express an EYFP reporter gene, replacing the luciferase readout implemented in earlier studies.^23^ This modification simplifies the screening and reduces costs, since EYFP requires no substrate addition. Furthermore, EYFP fluorescence enables a more direct estimation of 3CLpro inhibition than the non-linear luciferase readout. We first systematically optimized the circuit in designer HEK293T cells using flow cytometry. The system was adapted to plate reader measurements using designer HeLa cells engineered with the circuit and constitutively expressing ECFP, which functions as a cytotoxicity marker. These stably transduced designer cells improve inter-experimental consistency, reduce costs and labor compared with repeated transfection, can be expanded for multiple screens, and can be cryopreserved for long-term storage or distribution. Finally, we emphasized the importance of validating screening results using naive cells and flow cytometry to avoid false-positive observations.

Our findings demonstrate the potential of the screen while delineating its limitations, which stem from its optimization for rapid preliminary evaluation of numerous candidate compounds for downstream validation. Consequently, the screen may overlook pharmacodynamic parameters, including IC_50_ values and inhibition response curves, which require downstream characterization using appropriate methods such as molecular assays or functional evaluation using live virus.

In conclusion, synthetic gene circuits provide an advantage over protein engineering-based approaches. The circuit design is modular and tunable. For example, sensitivity can be adjusted by modifying circuit components, such as optimizing the CS domains or altering the output protein stability using protein degradation tags.^41^ Moreover, the output protein can be readily replaced with any reporter gene, enabling adaptation to various measurement platforms. This modularity also enables screening for drugs targeting additional proteases by replacing the protease encoded in module 1 and the corresponding CS domains in module 2.^23^ This approach could be extended to additional diseases, such as developing cancer modalities by targeting matrix metalloproteinases. Together, the proposed pipeline and designer cells establish an efficient, scalable, and physiologically relevant platform for the first-pass evaluation of antiviral drug candidates, thereby enhancing antiviral drug discovery.^14^^,^^16^

### Limitations of the study

The experimental pipeline consists of plate reader measurements and subsequent flow cytometry validations optimized to minimize labor and exclude false-positive results. This setup could be adapted to minimize the screening turnaround time but at the cost of increased labor. Yet, other methods may outperform our screening pipeline in specific contexts. Our approach is not optimized for precise IC_50_ determination, for which assays such as crystallography, FRET, mass spectrometry, and phenotypic screening may be more suitable.^9^^,^^10^^,^^11^^,^^12^ Moreover, enzyme-based screens, such as TAGS, provide superior sensitivity compared with our fluorescence-based screen and may be favorable for applications in which labor or cost constraints are not a limiting factor but detecting weak inhibitors is critical. In addition, fluorescence-based estimations are insufficient for the precise evaluation of drug-mediated cytotoxicity. For example, certain compounds may alter ECFP expression independently of their cytotoxic activity, either due to autofluorescence in the ECFP channel or by specifically inhibiting ECFP fluorescence. Therefore, dedicated cell viability assays must be performed in downstream evaluation steps. Finally, for applications requiring long-term use of designer cells, design modifications may be necessary to prevent transgene silencing, such as selecting robust promoters for circuit modules or incorporating DNA insulators to block repressive epigenetic modifications.^42^^,^^43^

## Resource availability

### Lead contact

Requests for further information, resources, and reagents should be directed to and will be fulfilled by the lead contact, Lior Nissim (lior.nissim@mail.huji.ac.il).

### Materials availability

Plasmids generated in this study have been deposited in Addgene. The Addgene IDs for all plasmids can be found in the key resources table.

### Data and code availability

• Raw data generated in the manuscript are included in the file Data S1 and can also be shared by the lead contact upon request.
• This paper does not report original code.
• Any additional information required to reanalyze the data reported in this paper is available from the lead contact upon request.

## Acknowledgments

This work was supported by the Israel Ministry of Science and Technology, the Israel Innovation Authority (IIA) Science Forefront call in genomics and artificial intelligence for drug discovery (grant no. 0002310; “Predicting multi-targeted antiviral drug candidates by machine learning and validating by synthetic biology”), and the 10.13039/501100003977Israel Science Foundation (ISF) KillCorona grants. Illustrations were created using BioRender.com. Graphs were generated with GraphPad Prism.

## Author contributions

Conceptualization, S.E., S.E.-A., and T.E.; data curation, S.E., S.E.-A., T.E., M.E., C.K., I.A., O.W., N.S., A.G., and L.N.; formal analysis, S.E., S.E.-A., T.E., M.E., C.K., I.A., O.W., N.S., A.G., and L.N.; funding acquisition, A.G. and L.N.; investigation, S.E., S.E.-A., T.E., M.E., C.K., I.A., O.W., N.S., A.G., and L.N.; methodology, S.E., S.E.-A., T.E., A.G., and L.N.; software, S.E.-A., I.A., O.W., N.S., and A.G.; supervision, A.G. and L.N.; visualization, S.E., C.K., S.E.-A., and L.N.; writing, T.L., S.E., S.E.-A., T.E., A.G., and L.N.

## Declaration of interests

The authors declare no conflict of interest in this study.

## Declaration of generative AI and AI-assisted technologies in the writing process

During the preparation of this work, the authors used Grammarly and ChatGPT-4o. These AI tools were employed to improve spelling, grammar, and readability. Edits were made by submitting each sentence separately. Subsequently, the authors reviewed and edited the content of each sentence as needed, and they take full responsibility for the content of the published article.

## STAR★Methods

### Key resources table

**Table:** 

REAGENT or RESOURCE	SOURCE	IDENTIFIER
**Chemicals, peptides, and recombinant proteins**		

FuGENE HD	Promega	E2312
OptiMEM	Thermo Fisher Scientific	31985–047

**Deposited data**		

Raw data	This paper	Data S1

**Experimental models: Cell lines**		

HEK-293T cells	ATCC	CRL-3216
HeLa cells	ATCC	CRM-CCL-2

**Recombinant DNA**		

pCMV-VSV-G	Stewart et al.	Addgene ID: 8454
psPAX2	Trono Lab Packaging and Envelope Plasmids (unpublished)	Addgene ID: 12260
pFUGW	Lois et al.	Addgene ID: 14883
pSE152	This study	Addgene ID: 240455
pTE3	This study	Addgene ID: 240456
pSE163	This study	Addgene ID: 240457
pTE5	This study	Addgene ID: 240460
pSE89	This study	Addgene ID: 240461
pSE92	This study	Addgene ID: 240462
pECFP	This study	Addgene ID: 24063

**Software and algorithms**		

LigPrep wizard	Schrödinger Release 2018-2	https://ir.schrodinger.com/press-releases/news-details/2018/Announcing-Schrdinger-Software-Release-2018-2-06-06-2018/default.aspx
Enamine HTS database	Enamine HTS Collection; accessed Dec 15, 2021	https://enamine.net/ (2021)
PDB repository	Berman et al.^44^	RCSB.org
Protein Preparation Wizard	Sastry^45^	https://ir.schrodinger.com release 2018-4
PROPKA	propka.ki.ku.dk	propka.org

### Experimental model and study participant details

#### Cell culture

Low-passage HEK-293T cells (CRL-3216, ATCC) and HeLa cells (CRM-CCL-2, ATCC) were cultured in Dulbecco’s modified Eagle medium (DMEM; 01-055-1A, Biological Industries) supplemented with 10% fetal bovine serum (FBS; 04-007-1A, Biological Industries), 1% MEM non-essential amino acids (MEM/NEAA; 01-340-1B, Biological Industries), 1% sodium pyruvate (03-042-1B, Biological Industries), and 100 units/mL penicillin with 0.1 mg/mL streptomycin (03-031-1B, Biological Industries). Cells were cultured at 37°C with 5% CO^2^.

### Method details

#### Plasmid construction

Plasmids used in this study were obtained from Addgene or constructed using conventional restriction cloning or Gibson assembly. Plasmid compositions are described below.

**Table:** Plasmid composition

Plasmid ID	Description	Composition	Plasmid type
**psPAX2**	Plasmid expressing the HIV-1 POL and GAG proteins (commercially obtained)	Lentiviral packaging component	Packaging
**pVSV-G**	Plasmid encoding the VSV-G envelope proteins used in conventional lentivirus production under the CMV promoter. (commercially obtained)	CMVp-VSV-G, Lentiviral envelope component	Envelope
**pSE152** (*Module 1*)	hUbC promoter regulating 3CLpro expression	pFuGW-hUbCp-3CLpro-SV40NLS	Expression
**pTE3** (*Module 2*, CS-Con X1)	Synthetic transcription factor with 1 copy of the 3CLpro consensus CS (AVLQSGFR) between GAL4BD and VP16AD	pFuGW-SSX1p-GAL4BD-1X3CLpro consensus CS- VP16AD-SV40NLS	Expression
**pSE163** (*Module 2*, CS-Gen X1)	Synthetic transcription factor with 1 copy of the 3CLpro synthetic general CS (VARLQSGF) between GAL4BD and VP16AD	pFuGW-SSX1p-GAL4BD-1X3CLpro synthetic general CS-VP16AD-SV40NLS	Expression
**pTE5** (*Module 2*, CS-Gen X4)	Synthetic transcription factor with 4 tandem repeats of the 3CLpro synthetic general CS (VARLQSGF) between GAL4BD and VP16AD	pFuGW-SSX1p-GAL4BD-4X3CLpro synthetic general CS- VP16AD-SV40NLS	Expression
**pSE89** (*Module 3*, G5p)	EYFP downstream to 5x GAL4 binding sites, regulated by the GAL4BD-CS-VP16AD synthetic transcription factor in *module 2*	pFuGW-G5p-EYFP	Expression
**pSE92** (*Module 3*, G14p)	EYFP downstream to 14x GAL4 binding sites, regulated by the GAL4BD-CS-VP16AD synthetic transcription factor in *module 2*	pFuGW-G14p-EYFP	Expression
**pECFP** (Stable ECFP expression)	hUbC promoter regulating ECFP expression	pFuGW-hUbCp-ECFP	Expression

#### Lentivirus production and transduction

##### Lentivirus production

Lentiviruses were produced by co-transfecting HEK293T cells in a 6-well plate format. Briefly, 6 μL of FuGENE HD (Promega, E2312) was mixed with 100 μL of OptiMEM (31985-047, Thermo Fisher Scientific) and combined with a mixture of three plasmids: 0.5 μg pCMV-VSV-G vector (Addgene #8454), 0.5 μg lentiviral packaging vector psPAX2 (Addgene #12260), and 1 μg lentiviral expression vector based on the pFUGW backbone (Addgene #14883). After a 20-min incubation of the FuGENE HD/DNA complexes at room temperature, HEK293T suspension cells were prepared and diluted to 2.5 × 10^6^ cells/mL in culture medium. 0.5 mL of the cell suspension (1.25 × 10^6^ cells) was added to each FuGENE HD/DNA complex tube, mixed thoroughly, and incubated for 5 min at room temperature. The mixture was then transferred to the designated well of a 6-well plate to a final volume of 1.6 mL and incubated at 37°C with 5% CO_2_. The medium was replaced 18 h post-transfection with 2.5 mL of fresh culture medium. The supernatant containing lentiviruses was collected 48 h post-transfection and filtered through a 0.45 μm syringe filter (Merck Millex, SLHVR33RS).

##### Lentivirus transduction

Stable cell lines engineered with the circuit were produced through three rounds of transduction. In the first round, cells were transduced with *Module 3* (GAL4p-EYFP). Filtered viral supernatants were used to transduce 2.5 × 10^5^ HEK293T or HeLa cells overnight in the presence of 8 μg/mL polybrene (Sigma, cat#107689) (See Cell Lines Composition, data file Data S1). The medium was replaced 48 h post-transduction, and cells were cultured for at least one week to allow recovery. The recovered cells were subsequently transduced with a lentiviral vector encoding *Module 2* (GAL4BD-CS-VP16AD) and cultured for an additional week until recovery. Finally, a third round of transduction was performed using a lentiviral vector encoding *Module 1* (3CLpro expression vector) (See Cell Lines Composition, data file Data S1).

To adapt HeLa cells for plate reader measurements, cells transduced with the circuit were further transduced with a lentiviral vector encoding ECFP (pECFP) (See Cell Lines Composition, data file Data S1).

#### Drug preparation

Nirmatrelvir and candidate small molecules were diluted in DMSO to create 100 mM stock solutions, which were stored at −20°C. Working solutions were prepared by diluting the stock solutions 1:50 in PBS to achieve a concentration of 2 mM, which was stored at 4°C. Appropriate volumes of the working solutions were added to wells to obtain the desired final drug concentrations.

**Table:** Dilution of small molecules to a 100mM stock solution

Compound	Barcode	MW	Amount mg	DMSO Vol (μL) for 100 mM dilution
17	Z1346455364	433.5	5.1	118
19	Z1395332235	361.4	5.2	144
20	Z140033838	444.5	5.2	117
21	Z1437386673	352.5	5.1	145
22	Z1438543103	345.5	5.1	148
25	Z1544077719	417.9	5.1	122
26	Z1569032362	345.5	5.2	151
27	Z1572466110	343.4	5.2	151
28	Z1574776365	438.5	5.2	119
29	Z1606803161	332.4	5.2	156
30	Z1633604757	314.4	5.1	162
31	Z1637696593	346.5	5.1	147
32	Z1675097879	387.5	5.1	132
33	Z1681933155	308.4	5.1	165
34	Z1688135161	344.5	5.1	148
35	Z1703261159	324.4	5.2	160
37	Z1731684610	334.4	5.1	153
38	Z1816257362	331.5	5.1	154
39	Z1816934508	338.4	5.1	151
40	Z1823362028	310.4	5.1	164
41	Z1823655466	444.6	5.1	115
43	Z1838367096	345.5	5.1	148
44	Z185315332	383.4	5.2	136
45	Z1942590281	342.5	5.2	152
46	Z195913386	368.4	5.2	141
47	Z2014723346	367.8	5.1	139
48	Z2027678255	389.5	5.2	134
49	Z2039966828	409.5	5.1	125
50	Z2058691671	370.4	5.1	138
51	Z208192278	360.2	5.2	144
53	Z2264638589	381.3	5.1	134
54	Z2362402924	335.4	5.2	155
55	Z2383437265	329.8	5	152
56	Z238739522	433.5	5.2	120
57	Z247217120	343.4	5.1	148
58	Z2606097246	377.5	5.2	138
59	Z2606109052	357.5	5.1	143
60	Z2642339806	325.4	5.1	157
61	Z2699320092	319.4	5.1	160
62	Z2700094476	320.5	5.2	162
63	Z27060073	394.5	5.2	132
64	Z2709355156	386.5	5.1	132
66	Z2734746451	327.4	5.1	156
67	Z2760952378	414.5	5.1	123
69	Z2766453790	305.4	5.1	167
70	Z2831553431	396.6	5.1	129
71	Z28602125	467.6	5.2	111
72	Z318836166	330.4	5.2	157
73	Z32748769	494.6	5.2	105
74	Z343569260	345.5	5.2	151
75	Z434427756	345.4	5.2	151
76	Z435812952	349.5	5.1	146
77	Z437024982	378.4	5.2	137
78	Z46325855	438.6	5.2	119
79	Z507822618	332.4	5.2	156
80	Z511845394	327.9	5.2	159
81	Z51905873	414.6	5.1	123
84	Z649153126	341.4	5.1	149
85	Z734235932	351.4	5.1	145
87	Z85918332	414.3	5.2	126
88	Z875397602	345.4	5.2	151
91	Z900486804	341.4	5.2	152
92	Z993401782	388.6	5.2	134
93	Z1187999982	322.4	5.1	158
94	Z1318178687	426.6	5.2	122
95	Z1447441058	349.8	5.1	146
96	Z1513513653	420.6	5.2	124
97	Z1544701527	342.5	5.1	149
99	Z1589742080	346.5	5.2	150
100	Z1597833238	349.5	5.1	146
101	Z1598156472	340.5	5.2	153
102	Z1638634322	328.4	5.2	158
104	Z1658740702	345.5	5.1	148
105	Z1665535243	377.4	5.1	135
107	Z1688137024	360.5	5.1	141
109	Z1895729207	348.5	5.1	146
110	Z1897140572	300.4	5.1	170
111	Z2020860964	306.5	5.1	166
112	Z2027692826	311.5	5.1	164
113	Z2264638596	393.4	5.2	132
114	Z2379491607	335.5	5.1	152
115	Z2465268599	316.4	5.1	161
116	Z2582964126	319.4	5.1	160
117	Z2591212195	335.5	5.2	155
118	Z2690309604	305.4	5.2	170
119	Z2690312219	305.4	5.1	167
120	Z2732692742	312.4	4.6	147
122	Z2737468456	304.4	5.2	171
123	Z2740576222	327.5	5.1	156
124	Z2740576223	304.5	5.2	171
125	Z2760968953	404.5	5.1	126
126	Z2761501603	329.4	5.1	155
127	Z2761502642	346.5	5.2	150
128	Z2761504115	328.5	5.1	155
132	Z977724862	358.5	5.1	142
133	Z977826924	344.5	5.1	148
134	Z977827526	358.5	5.1	142

#### Flow cytometry

To characterize fluorescent protein expression, the medium was removed, and cells were washed with PBS, detached with 0.5μL trypsin-EDTA, neutralized with an equal volume of medium, and centrifuged at 3000 rpm for 3 min to remove trypsin residues. The cell pellet was resuspended in PBS and analyzed using a cytoFLEX S flow cytometer (Beckman Coulter) with the appropriate laser setting. A minimum of 25,000 cells was recorded per sample. Data analysis was performed using Kaluza software (Beckman Coulter). The weighted median (WM) was calculated by multiplying the percentage of fluorescence-positive cells (% positive) by the median fluorescence of the positive cell population, yielding a value that correlates fluorescence intensity with the number of fluorescent-positive cells.^46^

#### Plate reader measurements

Cells were plated in 96-well plates (Greiner 96 Black Flat Bottom Fluotrac, #655090) at a density of 3250 cells per well and cultured in growth media supplemented with the appropriate compound or DMSO control. Each biological replicate consisted of three technical replicates per sample. Growth media, compounds, and DMSO were refreshed (Figure S1C). Before measurement, the supernatant was aspirated and replaced with 50 μL PBS. EYFP and ECFP fluorescence were quantified using a Synergy H1 microplate reader (Biotek) with the following settings: EYFP excitation 500 nm, emission 539 nm; ECFP excitation 435 nm, emission 480 nm; Gain = 100; 9 measurements per well. Fluorescence values were blanked and normalized to an empty well containing 50 μL PBS. Technical replicate values were averaged to determine the final fluorescence values for each biological replicate.

#### Cytotoxicity assays

Cells were plated in 96-well plates (Greiner 96 Black Flat Bottom Fluotrac, #655090) at a density of 5,000 cells per well in 50 μL of growth medium on Day 0 (D0), and an additional 100 μL of growth media was added to each well. On Day 2 (D2), the medium was replaced with fresh DMEM containing increasing volumes of DMSO to assess DMSO-induced cytotoxicity. Growth media and DMSO treatments were refreshed again on Day 4 (D4). On Day 6 (D6), the supernatant was aspirated and replaced with 50 μL PBS prior to fluorescence measurements. ECFP signals were quantified using a Synergy H1 microplate reader (BioTek) with the following settings: ECFP—excitation 435 nm, emission 480 nm; gain = 100; nine measurements per well. Fluorescence values were blank-subtracted and normalized to empty wells containing 50 μL PBS. Each biological replicate consisted of three technical replicates per sample, and technical replicates were averaged to determine final fluorescence values. Following fluorescence measurements, cells were collected from the plates, pooled across the three technical replicates, and counted using the Invitrogen Countess II FL Automated Cell Counter.

#### In silico screening

##### Molecule dataset preparation

The Enamine HTS database (2,159,632 compounds)^38^ was prepared for docking using the LigPrep wizard^47^ with the OPLS3e force field. Ionization and tautomeric states were generated with Epik.^48^ The states were generated at a target pH of 7 ± 2, limited to 32 stereoisomers per ligand.

##### Protein structure preparation

The structure of 3CLpro (PDB ID: 6LU7)^36^ was downloaded from the PDB repository (RCSB.org)^44^ and processed using Schrödinger’s ‘Protein Preparation Wizard’ (release 2018-4).^45^ Bond orders for amino acid residues and ligands were adjusted, after which hydrogen atoms, missing residues, and loops were added. Water molecules beyond 3 Å distance from the protein were removed. Hydrogen bond sampling, including adjustment of active site water molecule orientations, was performed using PROPKA (propka.ki.ku.dk) at pH 7. The structures were then refined using the OPLS3e force field restrained minimization, with the convergence of heavy atoms to an RMSD of 0.3 Å to relieve steric clashes.

##### Docking

The grid for the 3CLpro encompassed the inhibitor (N3) binding sites.^36^^,^^37^ 3CLpro forms a dimer, with each protomer consisting of three domains. The substrate-binding site is located in the cleft between domains I and II, and the catalytic dyad is formed by His41 and Cys145.^36^ Cys145 (S1120) was ionized to enable salt bridge formation.

The prepared Enamine compounds were docked to the grid using Glide standard SP docking settings^49^ and flexible ligand sampling. Docking scores were then used to evaluate ligand poses.

##### Docking filtration

To choose the top candidates for testing, we filtered the docking poses regarding two parameters.

(1) **Interaction filtration:** The output poses from the docking were filtered according to specific interaction criteria:
  • A salt bridge with Cys145.
  • A hydrogen bond with His163 or Glu166.
  • One of the following VdW interactions: His41, Met49, Phe140, Leu141, Asn142, His164, Met165, Asp187, Arg188, Glu189, Thr190.
(2) **Properties filtration:** For molecules that met the interaction criteria, a set of physicochemical properties was calculated using Schrödinger Maestro.^50^ A total score (TS) was calculated by assigning a value of 1 for meeting a criterion and 0 for not, with TS values ranging from 0 to 8 for each docking pose:
  • Docking score ≤ −6 Kcal/mol.
  • Number of contacts/VDW interactions ≥260.
  • Molecular charge >1.
  • H-bond acceptors (H_acc) ≤ 5.
  • H-bond donors (H_don) ≤ 2.
  • Log P (O/W) ≤ 2.
  • Buried Surface Area (BSA) > 800 Å^2^.
  • Rotatable bonds <6.

The criteria were applied to reduce the large number of docked molecules, many with multiple poses (Table included in the data file Data S1). The interaction energy is a key factor determining the probability of a molecule to bind its target. The larger number of VdW contacts is an additional favorable factor for binding, which is not entirely accounted for in fast SP docking. A positive charge, often mediated by an amine group, is essential for solubility, interaction with opposite charges on the target protein, and facilitating cellular uptake by binding negatively charged phosphate groups in its charged form and penetrating the membrane in its neutral form. The H-bond criteria balance solubility and binding through desolvation, as excess H-bond donors and acceptors increase the molecular solubility but limit the probability of leaving the aqueous environment. The lower logP criterion is aimed at lowering the probability that the molecule will be trapped in the membrane. The final two criteria relate to entropy. Higher BSA facilitates the release of water molecules from the protein-ligand complex into the surrounding solution, increasing Translational Entropy and reducing free energy. In contrast, a higher number of rotatable bonds leads to a loss of conformational entropy, favoring rigid molecules for binding.

### Quantification and statistical analysis

All graphs and statistical analyses were generated using GraphPad Prism software (GraphPad Software Inc., San Diego, CA, USA). Data are presented as the means of biological replicates, with individual dots on each bar representing replicate values and error bars indicating the standard error of the mean (SEM). Statistical significance was assessed using unpaired t-tests for comparison, as detailed in the figure legends. P-values <0.05 were considered statistically significant.

## Supplemental information

Data S1. Source data

Document S2. Article plus supplemental information
