# Supplementary material for: Optimized pipeline and designer cells for synthetic-biology-based high-throughput screening of viral protease inhibitors
Source: Cell Rep Methods. 2025 Aug 7;5(8):101139. doi: 10.1016/j.crmeth.2025.101139 (PMC12461586; doi:10.1016/j.crmeth.2025.101139)
Supplement: Document S1. Figures S1–S3 [file mmc1.pdf]

Cell Reports Methods, Volume *5*

Supplemental information

Optimized pipeline and designer cells for synthetic-biology-based high-throughput screening of viral protease inhibitors

Shlomi Edri, Shayma El-Atawneh, Tehila Ernst, Maayan Elnekave, Chaja Katzman, Tali Lanton, Ido Aldar, Omri Wolk, Noa Stern, Amiram Goldblum, and Lior Nissim

SUPPLEMENTARY DATA

Raw data generated in the manuscript, as well as cell line compositions, are included in the file Data S1 and can also be shared by the lead contact upon request.

Figure S1: Additional Controls and Protocol Optimizations in HEK293T, Related to Figure 1A-G

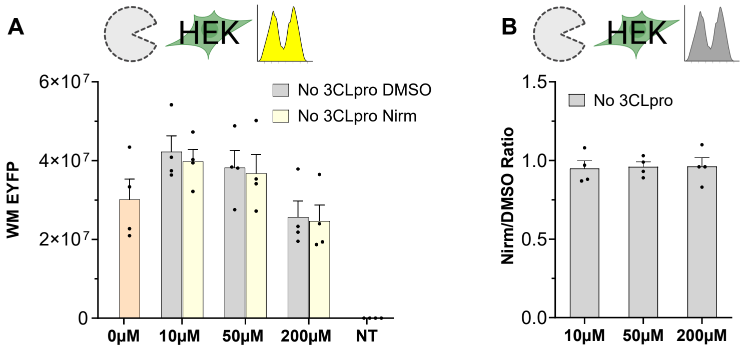

C

	Day 1	Day 2	Day 3	Day 4	Day 5	Day 6
**4 Days setup**	Plate cells with Media + Drug	-	Refresh Media + Drug	Measure		
**5 Days setup**	Plate cells with Media + Drug	-	Refresh Media + Drug	Refresh Media + Drug	Measure	
**6 Days setup**	Plate cells with Media + Drug	-	Refresh Media + Drug	-	Refresh Media + Drug	Measure

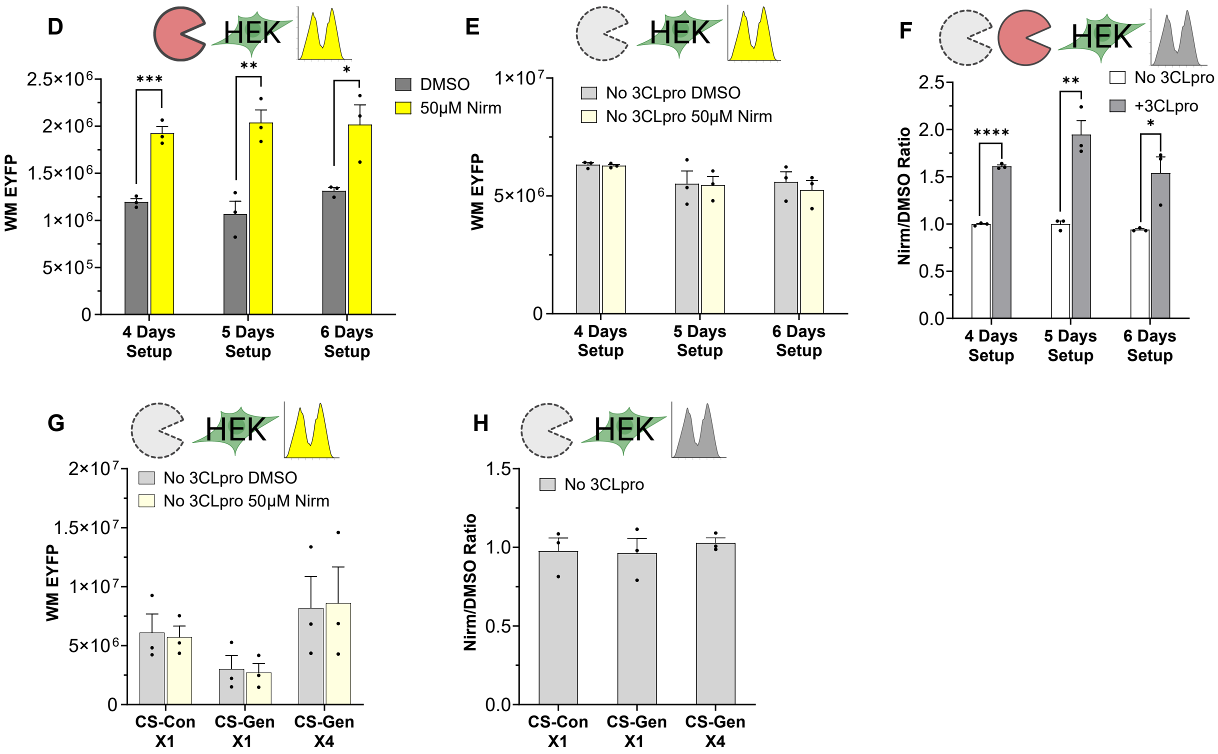

(A-B) EYFP output levels generated by control HEK293T cells engineered with *Module 2* and *Module 3*, but lacking 3CLpro (No 3CLpro), following Nirmatrelvir (Nirm) treatment. Results are shown as (A) The WM of EYFP fluorescence measured by flow cytometry and (B) Circuit sensitivity, defined as the ratio of fluorescence generated by a Nirm-treated sample to the fluorescence generated by the corresponding DMSO-treated control. **(C-F)** To optimize the experimental protocol, cells were treated with Nirm or DMSO for varying incubation periods and drug/media refreshment schedules, with flow cytometry measurements performed at different time points following treatment. (C) A table describing the different incubation periods and drug/media refreshments for each experimental setup examined. Results are shown as (D) The WM of EYFP fluorescence measured by flow cytometry in HEK293T cells engineered with all circuit modules (+3CLpro). Unpaired t-tests were performed to compare EYFP expression levels between Nirm-treated samples and their corresponding DMSO controls for each treatment protocol. (E) The WM of EYFP fluorescence was measured by flow cytometry in No 3CLpro HEK293T cells. (F) Circuit sensitivity in either +3CLpro or No 3CLpro cells. Unpaired t-tests were performed to compare the Nirm/DMSO ratio between +3CLpro or No 3CLpro samples. **(G-H)** Circuit output generated by No 3CLpro cells engineered with various cleavage sites (CS) in *Module 2*. No 3CLpro cells were transduced with *Module 2* variants in which the synthetic transcription factor contains either a single repeat of the consensus CS sequence AVLQSGFR (CS-Con x1), a single repeat of the synthetic general CS sequence VARLQSGF (CS-Gen x1), or four tandem repeats of the synthetic general CS sequence (CS-Gen x4). (G) The WM of EYFP fluorescence was measured by flow cytometry. (H) Circuit sensitivity in No 3CLpro cells.
Data are presented as the means of biological replicates, with individual dots on each bar representing replicate values. Error bars indicate the standard error of the mean (SEM); n = 3 or 4 biological replicates as indicated by the number of dots (*$p < 0.05$; **$p < 0.01$, ***$p < 0.001$, ****$p < 0.0001$).

Figure S2: Circuit Output in No 3CLpro HeLa cells, Related to Figure 1H-K

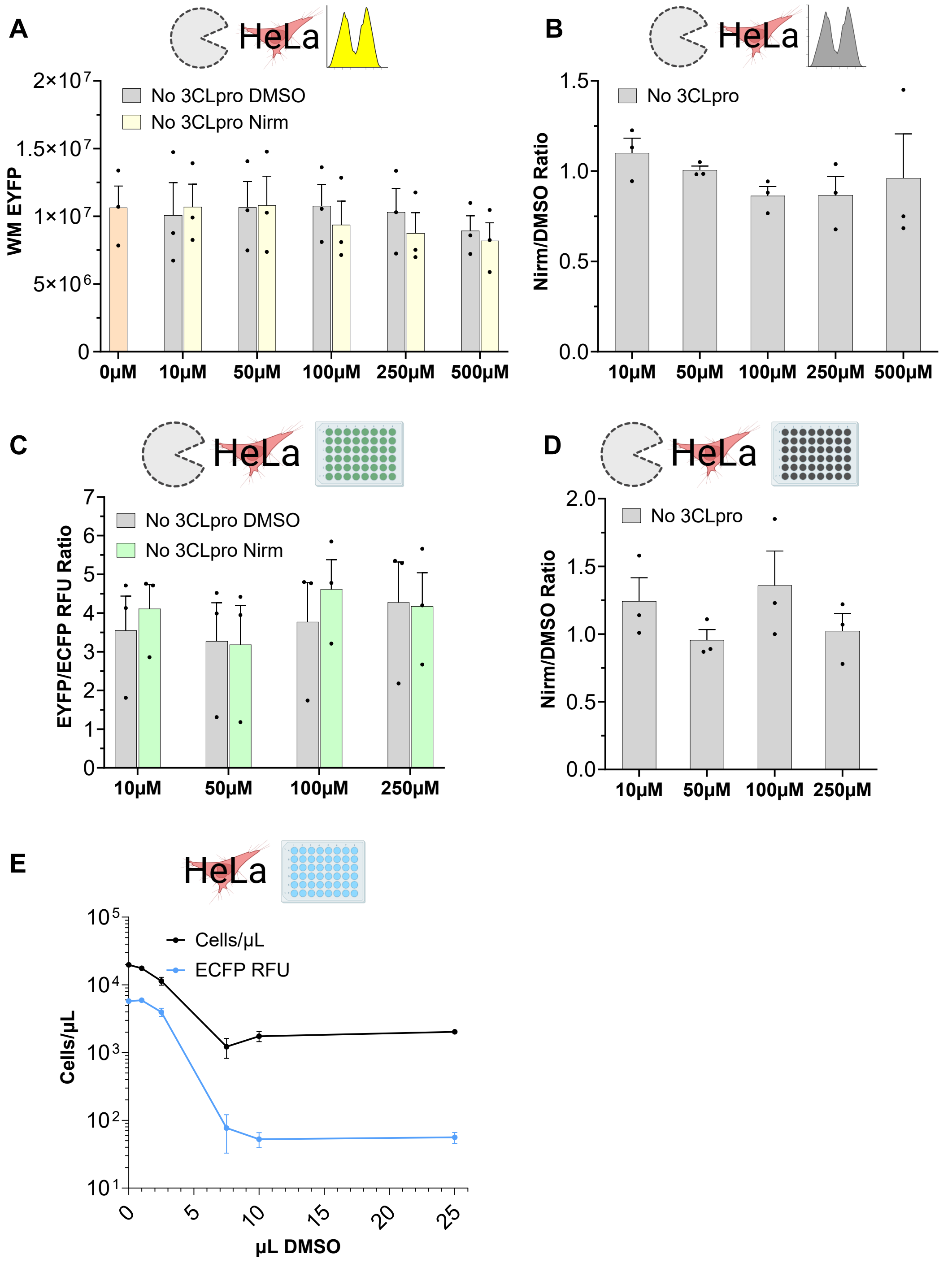

(A-B) EYFP output levels generated by control HeLa cells engineered with *Module 2* and *Module 3*, but lacking 3CLpro (No 3CLpro), following Nirmatrelvir (Nirm) treatment. Results are shown as (A) The WM of EYFP fluorescence measured by flow cytometry and (B) Circuit sensitivity, defined as the ratio of fluorescence generated by a Nirm-treated sample to the fluorescence generated by the corresponding DMSO-treated control. **(C-D)** Plate reader measurements of 3CLpro inhibition by Nirm in No 3CLpro HeLa cells. Results are presented as (C) the ratio of EYFP relative fluorescence units (RFU) to ECFP RFU and (D) Circuit sensitivity in plate reader measurements. **(E)** Correlation between ECFP levels and live cell number in HeLa designer cells. Results are presented as ECFP relative

fluorescence units (RFU) measured by a plate reader in each well, and live cell counts from the same wells as cells/μL.
Data are presented as the means of biological replicates, with individual dots on each bar representing replicate values. Error bars indicate the standard error of the mean (SEM); n = 3 biological replicates.

Figure S3: FACS Validations of 3CLpro Inhibition by Candidate Compounds, Related to Figure 2

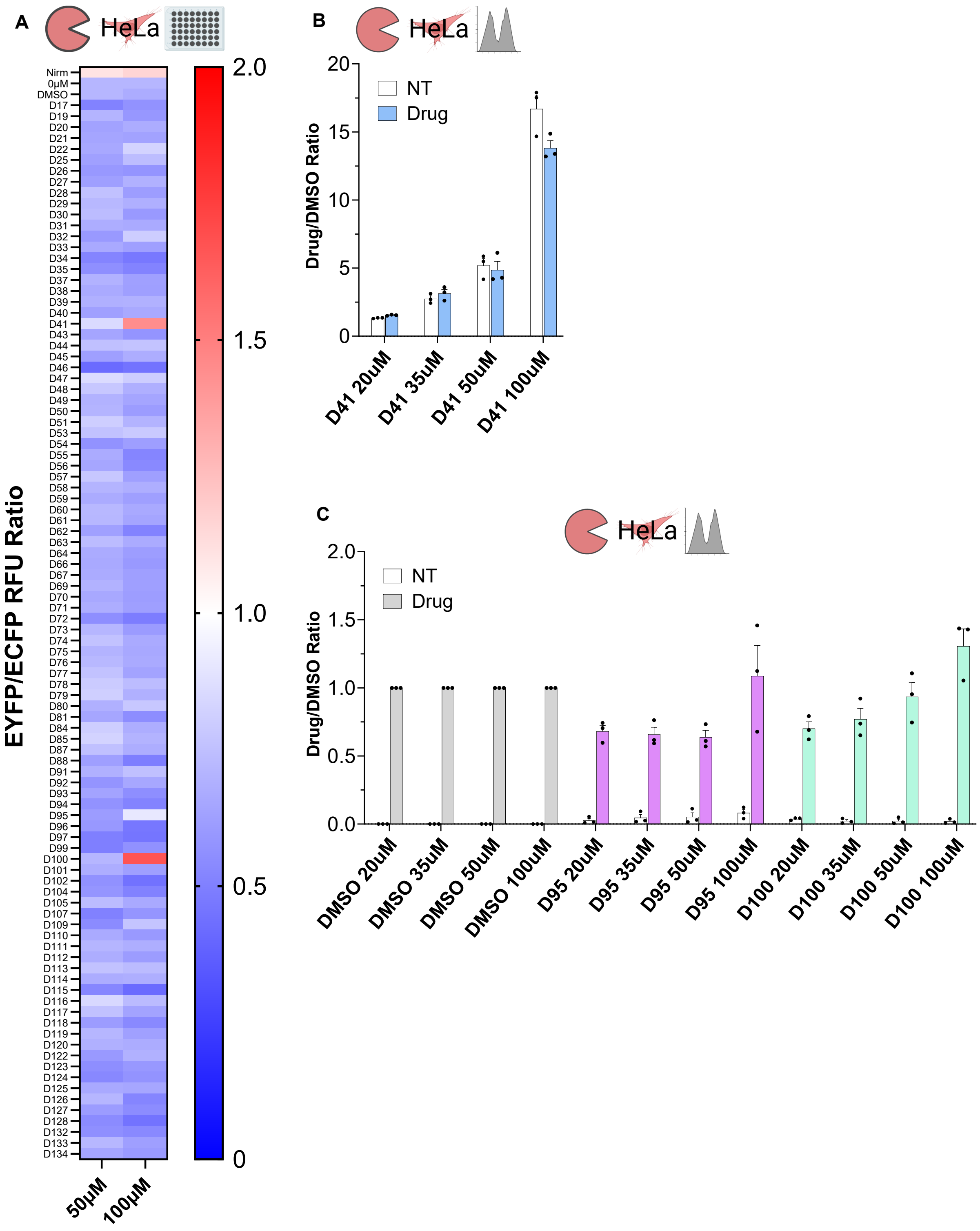

(A) Screening of 97 candidate 3CLpro inhibitory compounds in HeLa cells using plate reader measurements. Raw data results are presented as the ratio of EYFP/ECFP relative fluorescence units (RFU) values calculated for each sample. **(B-C)** The weighted median (WM) of EYFP fluorescence was measured by flow cytometry in HeLa cells, either naïve (NT) or engineered with all three circuit modules, following treatment with candidate compounds. The ratio of EYFP fluorescence generated by

a compound-treated sample to the EYFP fluorescence generated by the corresponding DMSO-treated control was then calculated to estimate 3CLpro inhibition by each compound. (B) Drug/DMSO EYFP output ratio for compound D41. (C) Drug/DMSO EYFP output ratio for DMSO controls, compound D95, and compound D100.

Data are presented as the means of biological replicates, with individual dots on each bar representing replicate values. Error bars indicate the standard error of the mean (SEM); n = 3 biological replicates.
